# Vacuolar ATPase Regulates Surfactant Secretion in Rat Alveolar Type II Cells by Modulating Lamellar Body Calcium

**DOI:** 10.1371/journal.pone.0009228

**Published:** 2010-02-16

**Authors:** Narendranath Reddy Chintagari, Amarjit Mishra, Lijing Su, Yang Wang, Sahlu Ayalew, Steven D. Hartson, Lin Liu

**Affiliations:** 1 Lundberg-Kienlen Lung Biology and Toxicology Laboratory, Department of Physiological Sciences, Oklahoma State University, Stillwater, Oklahoma, United States of American; 2 Department of Pathobiology, Oklahoma State University, Stillwater, Oklahoma, United States of America; 3 Department of Molecular Biology and Biochemistry, Oklahoma State University, Stillwater, Oklahoma, United States of America; University of Giessen Lung Center, Germany

## Abstract

Lung surfactant reduces surface tension and maintains the stability of alveoli. How surfactant is released from alveolar epithelial type II cells is not fully understood. Vacuolar ATPase (V-ATPase) is the enzyme responsible for pumping H^+^ into lamellar bodies and is required for the processing of surfactant proteins and the packaging of surfactant lipids. However, its role in lung surfactant secretion is unknown. Proteomic analysis revealed that vacuolar ATPase (V-ATPase) dominated the alveolar type II cell lipid raft proteome. Western blotting confirmed the association of V-ATPase a1 and B1/2 subunits with lipid rafts and their enrichment in lamellar bodies. The dissipation of lamellar body pH gradient by Bafilomycin A1 (Baf A1), an inhibitor of V-ATPase, increased surfactant secretion. Baf A1-stimulated secretion was blocked by the intracellular Ca^2+^ chelator, BAPTA-AM, the protein kinase C (PKC) inhibitor, staurosporine, and the Ca^2+^/calmodulin-dependent protein kinase II (CaMKII), KN-62. Baf A1 induced Ca^2+^ release from isolated lamellar bodies. Thapsigargin reduced the Baf A1-induced secretion, indicating cross-talk between lamellar body and endoplasmic reticulum Ca^2+^ pools. Stimulation of type II cells with surfactant secretagogues dissipated the pH gradient across lamellar bodies and disassembled the V-ATPase complex, indicating the physiological relevance of the V-ATPase-mediated surfactant secretion. Finally, silencing of V-ATPase a1 and B2 subunits decreased stimulated surfactant secretion, indicating that these subunits were crucial for surfactant secretion. We conclude that V-ATPase regulates surfactant secretion via an increased Ca^2+^ mobilization from lamellar bodies and endoplasmic reticulum, and the activation of PKC and CaMKII. Our finding revealed a previously unrealized role of V-ATPase in surfactant secretion.

## Introduction

Lipid rafts are specialized microdomains on the plasma membrane and subcellular membranes. Lipid rafts are highly enriched in saturated lipids including sphingolipids, and cholesterol, and specialized groups of proteins such as those which are acylated (Src kinases), and myristoylated/palmitoylated proteins (flotillins). Cholesterol depletion results in decreased association of raft proteins and ultimately their associated functions. Lipid rafts are implicated in exocytosis [1], [2], endocytosis [3], signal transduction [4], membrane trafficking [5], bacterial entry [6], and virus budding [7]. They are also associated with a number of metabolic diseases including Alzheimer's [8].

The cuboidal alveolar type II cells synthesize, store and secrete lung surfactant, a lipid-rich surface active substance. Lung surfactant lowers the surface tension and prevents the collapse of alveoli. The secretion of surfactant is a relatively slow process when compared to neurotransmitter release. Lung surfactant secretion is a highly regulated process. Our laboratory has earlier reported that SNAP-23, syntaxin 2, NSF and α-SNAP are critical for lung surfactant secretion [9], [10]. SNAP-23 associates with lipid rafts to a greater extent in comparison with syntaxin 2 and VAMP-2. Cholesterol depletion not only drastically reduces surfactant secretion but also the fusion of lamellar bodies with the plasma membrane [1]. Knock-down of flotillin-2, a lipid raft marker that is present on the lamellar body and plasma membranes, decreases surfactant secretion [11].

Lipid rafts contain distinct proteins. The proteomic profile of lipid rafts would help to uncover the protein machinery for exocytosis considering importance of lipid rafts in surfactant secretion. Proteomic studies of lipid rafts have previously been undertaken in T-cells [12], [13], human endothelial cells [14], mouse spermatosa [15], human smooth muscle cells [16], rat intestinal mucosal cells [17], exocrine pancreatic cells [18], and HL-60 cells [19]. These studies have indicated that lipid rafts are composed of the proteins involved in phosphorylation, cytoskeletal rearrangements, exocytosis, cell cycle and signal transduction [20].

Vacuolar ATPases (V-ATPases) are multi-subunit enzymes that drive the movement of protons using the energy of ATP hydrolysis [21]. They are present on intracellular organelles including endosomes, lysosomes, secretory granules and synaptic vesicles, and also mediate the acidification of these organelles. Organellar acidification is crucial for the dissociation of ligand-receptor complexes, the processing of secretory proteins and accumulation of neurotransmitters. V-ATPases also exist on the plasma membranes in some specialized cells such as macrophages, neutrophils, kidney intercalated cells and osteoclasts. Extracellular acidification is required for bone resorption, urinary acidification, and the maintenance of intracellular pH. The mutations in genes coding for V-ATPase subunits contribute to a number of diseases [22]–[24].

Lamellar bodies are secretory granules that store lung surfactant in type II cells. They have lysosomal properties and maintain an internal acidic milieu owing to the presence of V-ATPases [25]. The acidic lamellar body pH is crucial for surfactant protein B and C processing [26] and the package of surfactant lipids [27]. However, the role of V-ATPase in surfactant secretion is unknown.

In this study, we attempted to identify new components in type II cell lipid rafts using mass spectrometry. Our results revealed a number of proteins involved in energy metabolism, cytoskeletal re-arrangement, cell proliferation and pH regulation. We further studied the role of one of the identified enzyme, V-ATPase in lung surfactant secretion and underlying mechanisms by which they regulate the secretory process.

## Materials and Methods

### Reagents

Horseradish peroxidase (HRP)-conjugated goat anti-rabbit antibody and protein molecular mass markers and all the reagents and chemicals used for 2-D gel electrophoresis were purchased from Bio-Rad (Hercules, CA). HRP-conjugated goat anti-mouse antibody was from Jackson Immunologicals (West Grove, PA). Monoclonal mouse anti-flotillin-1 antibody was from BD Biosciences (San Jose, CA), polyclonal rabbit anti-V-ATPase a1 (H-140) and monoclonal mouse anti-V-ATPase B1/2 were from Santa Cruz Biotechnology (Santa Cruz, CA). Monoclonal mouse anti-Na^+^-K^+^ ATPase α1-subunit antibody was from Upstate Biotechnology (Lake Placid, NY). Bafilomycin A1 (Baf A1) was from LC Laboratories (Woburn, MA). Enhanced Chemiluminescence (ECL) detection system was from GE HealthCare (Piscataway, NJ). Methyl-^3^H-Choline chloride and ^45^CaCl_2_ were from PerkinElmer (Waltham, MA).

### Alveolar Type II Cell Isolation

Type II cells were isolated from Sprague-Dawley male rat lungs as described earlier [1]. Purity and viability of the cells were greater than 90 and 95%, respectively. The Oklahoma State University Animal Care and Use Committee approved all the animal procedures used in this study.

### Isolation of Lipid Rafts

Lipid rafts were isolated exactly as described earlier [1]. Freshly isolated type II cells were washed once with MEM and then with MBS (25 mM MES and 150 mM NaCl, pH 6.5) buffer twice. The cells were lysed in ice-cold 1% Triton X-100 (v/v) and incubated on ice for 45 min. For cholesterol depletion, type II cells were lysed with 0.5% Triton X-100 and 0.5% (w/v) saponin. Equal amounts of proteins (∼3 mg) in 600 µl of lysis buffer were mixed with an equal volume of 80% sucrose and laid at the bottom of ultracentrifuge tubes. Then, 1200 µl of 30% and 5% sucrose were laid on the top of 40% sucrose. The gradients were centrifuged at 200,000×g for 18 hrs. Eight fractions were collected from top to bottom of the gradients in the following manner: fractions 1 and 2, 600 µl each; fractions 3, 4, and 5, 400 µl each; fractions 6 and 7; 600 µl each. Fraction 8 was the pellet dissolved in 600 µl of lysis buffer. Equal volumes of the fractions were probed for the raft marker protein, flotillin-1 and non-raft marker protein, Na^+^-K^+^ ATPase. Lipid rafts were collected from the interface between 5% and 30% sucrose gradients *i.e.*, fraction 3. The raft fractions were diluted four times with MBS buffer, followed by centrifugation at 100,000×g for 30 min. The pellet was resuspended in MBS buffer and stored at −80°C until proteomic analysis. We consistently obtained 80–125 µg of raft proteins from 25 million type II cells.

### Western Blotting

Equal volumes of various fractions were immunoblotted for flotillin-1 and Na^+^-K^+^ ATPase exactly as described in [1]. For determining the association of V-ATPase subunits with lipid rafts, equal volumes of lipid raft fractions were probed with mouse monoclonal anti-V-ATPase B1/2 antibodies and rabbit polyclonal anti-V-ATPase a1 (1∶1000), followed by HRP-conjugated goat anti-mouse and goat anti-rabbit antibodies (1∶2500) before visualizing the proteins by ECL system.

To determine the localization of V-ATPase subunits a1 and B1/2, equal amounts of proteins isolated from lung plasma membranes, lamellar bodies, type II cells and lung homogenates were immunoblotted exactly as described above. Lung plasma membranes and homogenates were isolated as described in [1].

### 2-Dimensional (2-D) Gel Electrophoresis

Lipid raft fractions were first cleaned up using the ReadyPrep 2-D cleanup kit and then resuspended in 2-D rehydration/sample buffer (8 M Urea, 2% CHAPS, 50 mM DTT, 0.2% Bio-Lyte 3/10 ampolyte and 0.002% bromophenol blue). Immobilized pH gradient (IPG) strips (pH 3–10) were passively hydrated with the protein samples overnight at room temperature. The proteins were separated on the first dimension by isoelectric focusing (IEF) at room temperature for 12–16 hrs and 40–60,000 Vhrs. Following IEF, the IPG strips were equilibrated in SDS-PAGE equilibration buffer I [6 M Urea, 0.375 M Tris-HCl, pH 8.8, 2% SDS, 20% glycerol and 2% (w/v) DTT] for 10 min and then with buffer II (equilibration buffer I plus 2.5% (w/v) iodoacetaminde and without DTT] for an additional 10 min. The proteins were further separated on precast 8–16% polyacrylamide gradient gels based on their molecular masses. The gels were fixed with 40% (v/v) methanol and 10% (v/v) acetic acid for 1 hr, washed with sterile water two times (10 min each) and stained for overnight in staining buffer [0.08% (w/v) Coomassie brilliant blue G250, 1.6% (w/v) ortho-phosphoric acid, 8% (w/v) ammonium sulphate and 20% (v/v) methanol]. The gels were then destained with 1% acetic acid several times until the Coomassie stain particles were removed. The gels were scanned and proteins spots excised for trypsinolysis and mass spectrometry.

### Matrix-Assisted Laser Desorption/Ionization-Time Of Flight (MALDI-TOF) Mass Spectrometry

The gel spots were first destained with 50% acetonitrile, then with 100% acetonitrile and 100 mM ammonium bicarbonate (ABC). The gels were subsequently rehydrated with freshly made reducing buffer (10 mM DTT and 25 mM ABC) for 1 hr at 56°C. The proteins were subjected to alkylation for 1 hr (55 mM iodoacetamide in 25 mM ABC) and then to dehydration and rehydration with 100% and 50% acetonitrile for an additional 1.5 hrs. Subsequently the proteins were digested with trypsin (8.3 µg/ml in 25 mM ABC) for 4 hrs at 37°C. The trypsinolytic peptides were then extracted with 0.1% trifluoroacetic acid, spotted onto MALDI plates and overlaid with freshly made matrix (5 mg/ml of alpha-cyano-4-hydroxycinnamic acid). For calibration, peptide standards were spotted in adjacent wells of the plate. Mass spectra were collected using Voyager DE-PRO mass spectrometer (Applied Biosystems) operated in the reflector mode to yield 100 ppm mass accuracy.

### Database Searching

Monoisotopic peptide masses were used to search mammalian and rodent protein sequence databases using the MASCOT software (Matrix Science Ltd., London). The proteins were identified by the probability based MOlecular Weight SEarch (MOWSE). All the proteins whose Mascot scores were greater than the Mascot thresholds were considered as positive hits. Peaks due to keratin contamination were excluded from database searching. Search parameters included: one missed cleavage per peptide, and the variable modifications carbamidomethyl and propionamide of cysteines and oxidation of methionine. The peptide mass tolerance was set at ±100 ppm, and peptide charge state of 1+. All matching spectra were manually reviewed.

### Lung Surfactant Assay

Lung surfactant secretion was assayed by monitoring phosphatidylcholine (PC) released from type II cells as previously described [1]. Type II cells were labeled with [^3^H] choline overnight. The dishes were then washed to remove unattached cells. The cells were treated with 20 nM Baf A1 for 1 hr. One set of dishes were taken out for determining the PC secretion during this time (zero time). The cells were stimulated with a combination of secretagogues (100 µM ATP, 0.1 µM PMA and 10 µM terbutaline) for an additional 2 hrs. Media and cells were collected and lipids were extracted. Surfactant secretion was expressed as (^3^H-labeled PC in medium/^3^H-labeled PC in medium plus cells) ×100%. Net secretion was obtained by subtracting the zero time value.

To examine the role of extracellular Ca^2+^ in V-ATPase-mediated lung surfactant secretion, overnight-cultured cells were washed and incubated with 20 nM Baf A1 for 1 hr in the buffer [in mM: 118 NaCl; 5 KCl, 25 NaHCO_3_; 1 KH_2_PO_4_; 1 MgCl_2_; 1 EGTA; 10 glucose; 30 HEPES (pH 7.4)] with and without 2 mM Ca^2+^. The cells were assayed for PC secretion as before.

To study the role of endoplasmic reticulum (ER) and intracellular Ca^2+^ in the Baf A1-induced PC secretion, the cells were pre-treated with 100 µM thapsigargin or 50 µM BAPTA-AM for 15 min and then with 20 nM Baf A1 for 1 hr. The zero time value was determined at this time point. The secretion was measured after additional 2-hr incubation.

To determine whether protein kinase C (PKC) and Ca^2+^/Calmodulin-dependent Kinase II (CaMKII) were involved in the Baf A1-mediated secretion, overnight cultured type II cells were treated with 20 nM Baf A1 and 100 nM staurosporine or 10 µM KN-62 for 1 hr and the zero time value was measured. The cells were incubated for additional 2 hrs and then assayed for surfactant secretion.

### ^45^Ca^2+^ Release from Isolated Lamellar Bodies

All procedures for lamellar bodies were performed at 4°C unless stated. Lamellar bodies were isolated from rat lungs [1]. Ca^2+^ sequestration and release experiments were done as described [28]. In brief, the isolated lamellar bodies (40–50 µg) were resuspended in 10 mM Tris-HEPES buffer (pH 7.5) containing 0.5 mM EGTA and 0.24 M sucrose. Ca^2+^ loading into lamellar bodies was initiated by the addition of 0.65 mM CaCl_2_ containing ^45^Ca^2+^ (66 cpm/pmol). The lamellar bodies were then incubated for 30 min at 37°C. Later, the lamellar bodies were diluted with equal volumes of buffer without (control) and with 10 nM Baf A1 and incubated at 37°C for additional 10 min. The reaction was immediately stopped by filtering the lamellar body suspension through a nitrocellulose membrane (pore size: 0.45 µm). The filters were washed with 10 ml of ice-cold Tris-HEPES buffer containing 0.24 M sucrose and 5 mM EGTA. The filters were subjected to scintillation counting.

### Labeling of Lamellar Body Surfactant

Adult Sprague-Dawley rats were intraperitoneally injected with 2 µl [^3^H] choline chloride per gram body weight (concentration: 0.25 µCi/µl). Following overnight labeling, lamellar bodies were isolated and treated exactly as described above for the Ca^2+^ release studies. At the end of incubation, the lamellar bodies were filtered and subjected to liquid scintillation counting.

### Quinacrine Staining

Type II cells were plated at a density of 0.5–1×10^6^ cells in 35 mm^2^ dishes. Following overnight culture in DMEM (supplemented with 10% FBS, non-essential amino acids, penicillin and streptomycin), the dishes were washed to remove unattached cells. Freshly made quinacrine was added to media at a final concentration of 10 µM. Lamellar body staining was examined at room temperature within 1 min upon the addition of the dye using a Nikon Eclipse TE2000-U inverted fluorescence microscope with a Plan Flour, ELWD 40×/0.6 objective lens. The images were captured with a CoolSnap CCD camera (Photometrics, Tucson, AZ) and the Metavue software (Molecular Devices Co; Downingtown, PA).

For determining the effects of lung surfactant secretagogues on the lamellar body pH, overnight cultured type II cells were stimulated with lung surfactant secretagogues for various times. At the end of incubation, the cells were stained with 10 µM quinacrine for one minute. The media was immediately decanted and the cells fixed with 4% paraformaldehyde. The slide was examined by fluorescence microscopy. We included unstimulated control cells for each of the time points to prevent bias with respect to loading and quenching of the dyes. The microscopic fields were chosen at random and at least 2–3 fields with about 8–10 cells in each field were selected for further analysis. The time lapse between capturing the images under stimulated and unstimulated conditions was ≤5 min. During this time frame, we did not observe any quenching of the dye. Identical exposure settings were used for capturing images for each time-point. The images were captured with a 40× objective lens. In some of the experiments, the order of capturing images between the control and treated cells was reversed to check for differences that might have resulted from the timing of image captures. In our hands, the order of capturing images did not reveal any differences when captured within the time frame. The intensity of quinacrine was quantitated using the Metavue software and expressed as arbitrary units per cell.

### Immunostaining

Overnight cultured type II cells on glass coverslips were stimulated with a combination of secretagogues (100 µM ATP, 0.1 µM PMA and 10 µM terbutaline) for 2 hrs. The cells were then fixed with 4% formaldehyde for 30 min at room temperature. The cells were permeabilized with 1% Triton X-100 for 20 min and then blocked with 10% fetal bovine serum for 1 hr. Mouse anti-V-ATPase B1/2 and anti-LB 180 antibodies were added at a dilution of 1∶50 and 1∶500, respectively and incubated overnight at 4°C. The cells were washed and incubated with Cy3-conjugated goat anti-mouse antibodies (1∶250) for 1 hr at room temperature. The cells were washed, mounted onto glass slides and examined with using a Nikon Eclipse E600 fluorescence microscope with a Plan Flour, ELWD 40×/0.75 objective lens. The images were captured with a Photometrics CoolSnap CCD camera and the Metavue software.

### Construction of Adenoviral Vectors

Silencing of V-ATPase subunits was achieved by using our recently developed novel adenoviral vector expressing 4 small interfering RNAs (siRNA), which was constructed exactly as described before [29]. The four small interfering RNA (siRNA) sequences for a1 subunit were: 5′- GTTAGAAGATGTGAAGAAATG-3′ (321–341), 5′-CCAGGAAGCTCTAAAGCGAAA-3′ (500–520), 5′- CTCCTTCAAGATGAAGATGTC-3′ (1760–1780), and 5′ GATGAAGATGTCAGTTATTCT-3′ (1769–1789). The siRNA sequences for B2 subunit were: 5′-GGATATGCTTGGTCGAGTATT-3′ (388–408), 5′- GGTAGAAATGGCTCTATTACC-3′ (1040–1060), 5′-GCTGCACAACAGACAGATTTA-3′ (1159–1179), and 5′- GAGTACCCTGAGCGAATTTTA-3′ (1504–1524). A vector containing irrelevant sequences was used as a control [29]. The viruses were labeled as follows: *AdVoa1* for a1 subunit; *AdV1B2* for B2 subunit; and *AdCon* for irrelevant control sequences.

### Knockdown of V-ATPase a1 and B2

Freshly isolated type II cells (day 0) were transduced with equal doses (100 multiplicity of infection or MOI) of adenoviruses and cultured in plastic dishes in DMEM supplemented with 10% FBS and antibiotics. Following overnight culture unattached cells were removed by washing the cells with fresh media. The cells were cultured for 5 more days. Media was changed every 48 hours. Cell lysates were collected after 5 day culture. Equal amounts of proteins were immunoblotted for a1 and B2 subunits. Equal loading was confirmed by reprobing blots with antibodies against GAPDH. For surfactant secretion, an air-liquid culture system was used to maintain type II cell phenotype as described earlier [11]. The cells were transduced with adenoviruses at a MOI of 100 on day 2 of culture. After culturing 5 more days, surfactant secretion was assayed.

### *MTT assay*

The viability of the cells following V-ATPase inhibition was monitored using MTT assay exactly as described [1].

### Statistical Analysis

All of the surfactant assays and quinacrine experiments were repeated with at least 3 independent cell preparations. ANOVA, followed by Newman-Keuls Multiple Comparison tests or Student *t* test were used for statistic analysis. A value of P<0.05 was considered as significant.

## Results

### Proteomic Analysis

**Figure 1:**
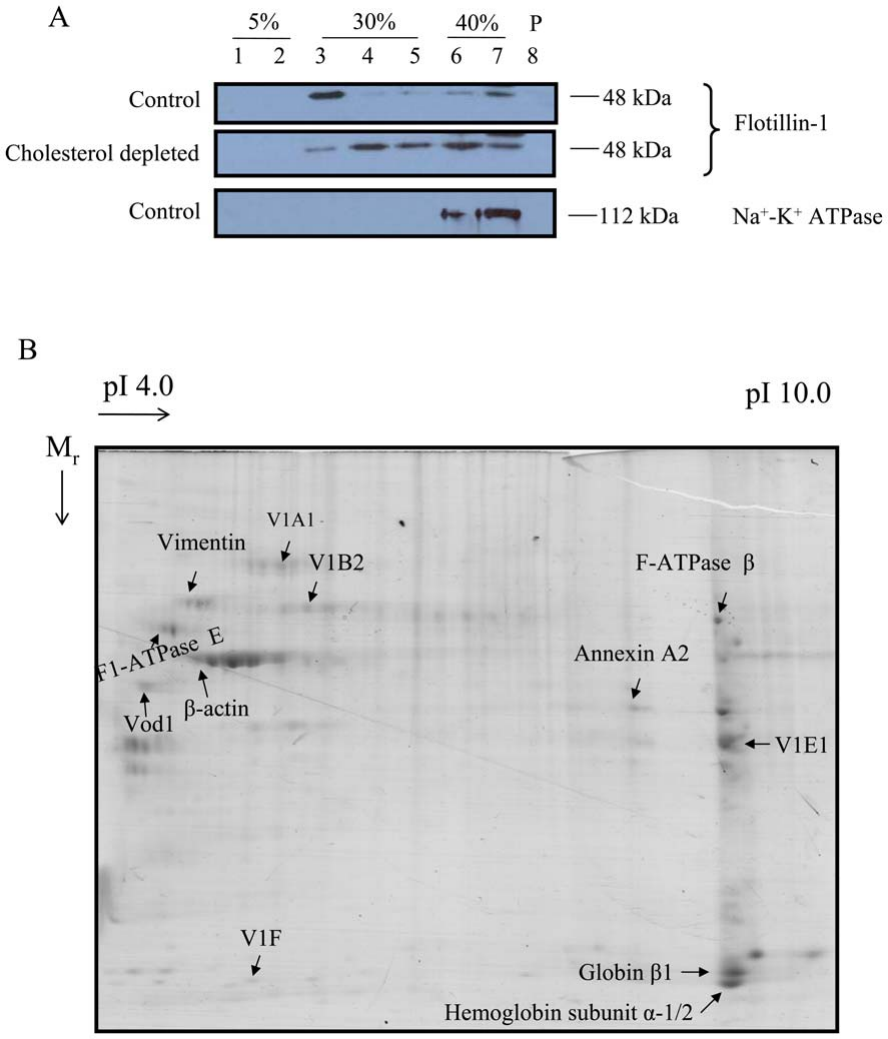
2-D gel electrophoresis of alveolar type II cell lipid rafts. (A) Confirmation of lipid rafts isolated from type II cells: Freshly isolated type II cells were lysed in the presence of 1% Triton X-100 (control) or 0.5% Triton X-100 and 0.5% saponin (cholesterol depleted) at 4°C for 45 min. Later, the lysate was subjected to a sucrose gradient centrifugation. Seven Fractions were collected from the top. The pellet was dissolved in lysis buffer (fraction P). The fractions were immunoblotted for flotillin-1, a raft marker protein and Na^+^-K^+^ ATPase, a non-raft marker protein. (B) 2-D gel electrophoresis: Lipid rafts proteins were subjected to 2-D gel electrophoresis. Later, the gels were stained with Coomassie blue. Shown is a representative of 3 runs.

We isolated lipid rafts from alveolar type II cells and performed proteomic analysis to identify proteins present in lipid rafts. Alveolar type II cells were solubilized with 1% Triton X-100 and subjected to a sucrose gradient centrifugation as previously described [1]. The isolated rafts were enriched in flotillin-1, a lipid raft marker but excluded Na^+^-K^+^-ATPase, a non-raft marker protein (Fig. 1A, fraction 3). Additionally, the association of flotillin-1 was cholesterol-dependent, which confirmed the lipid raft isolation protocol [1]. Raft proteins (500–600 µg) were separated on 2-D PAGE gels, stained, and identified by trypsinolysis, mass spectrometry, and database searching. In addition to the rat database, which is relatively small, we also searched the human and mouse databases for the identification of the proteins. Key protein identifications are shown in Fig. 1B and the metrics of their proteomics identifications are summarized in Table 1. The list included cytoskeletal proteins, V-ATPases (V1 subunits, A, B2, E1 and F, and Vo subunit d1), phospholipid binding proteins and some mitochondrial enzymes. Three proteins (SPFH domain family member 2, prohibitin, and caveolin-1β) listed in Table 1 were not labeled in Fig. 1B because they were identified in the other runs. Due to its importance in lamellar body acidification, we chose V-ATPase for further studies.

**Table 1:** Proteomic profile of lipid rafts isolated from alveolar type II cells.

Protein	NCBI Accesion #	M_r (kDa)_	pI	Peptides matched/Searched	Sequence covered	Mascot score/Mascot threshold
**Vacuolar Acidification**						
V-ATPase, V1 subunit A,	AAC52410	68	5.46	23/103	36	97/67
V-ATPase, V1 subunit B2	AAC52411	57	5.57	14/79	27	155/55
V-ATPase, Vo subunit D1	Q5M7T6	40	4.89	8/23	26	79/55
V-ATPase, V1 subunit E1	Q6PCU2	26	8.44	12/52	43	84/55
V-ATPase, V1 subunit F	BAB24692	13	5.52	8/35	63	97/55
**ATP synthesis**						
F-ATPase subunit β	1MABA	53	8.73	19/48	37	151/55
F-ATPase, subunit E	1MABB	49	5.11	11/26	34	97/63
**Cytoskeletal Associated**						
β –actin	ATRTC	42	5.29	10/14	28	113/55
Vimentin	P31000	54	5.06	22/96	45	126/55
SPFH domain family, member 2	Q8BFZ9	38	5.37	10/10	25	157/63
**Cell Cycle and repair**						
Prohibitin	AAH72518	30	5.57	8/13	24	84/63
**Ca^2+^-dependent membrane binding**						
Annexin A2	NP_06370	39	7.53	19/50	52	155/55
**Signal transduction**						
Caveolin 1β	AAL33580	17	4.97	7/19	31	76/55
**Miscellaneous**						
Globin β1	AAB30298	16	7.98	8/46	58	81/55
Hemoglobin subunit α−1/2	HBA_RAT	15	7.93	5/15	50	70/55

### V-ATPase Subunits Are Associated with Lipid Rafts

**Figure 2:**
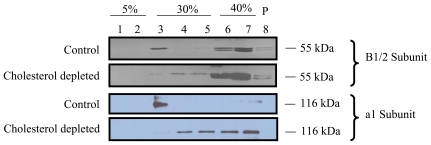
Association of V-ATPase subunits with lipid rafts. Type II cells were lysed in 1% Triton X-100 (control) or 0.5% Triton X-100+0.5% saponin (cholesterol depleted). Later, the lysates were subjected to raft isolation and various fractions were collected. Equal volumes of fractions were immunoblotted for V-ATPase B1/2 and a1 subunits.

We used Western blot analysis to confirm the association of V-ATPase with lipid rafts. V-ATPase is composed of a peripheral V1 domain and an integral membrane Vo domain. We selected two subunits, one from V1 (B subunit) and another from Vo (a1 subunit) based on the availability of suitable antibodies. The antibody for B subunit recognizes both B1 and B2 isoforms. Each fraction following raft isolation was examined for the presence of V-ATPase B1/2 and a1 subunits. As shown in Fig. 2, B1/2 and a1 subunits were present in the raft fraction (fraction 3). Furthermore, the depletion of cholesterol resulted in the dissociation of both subunits with the lipid rafts. It was noted that B1/2 subunits were partially associated with lipid rafts while most of a1 subunit was co-localized with the lipid raft fraction. This is probably due to the disassociation of B1/2 subunits from the Vo domain during isolation since B1/2 subunits are peripheral subunits. It is also possible that some B1/2 subunits are associated with cytoskeleton or non-lipid raft proteins. Our results indicated a genuine association of B1/2 and a1 subunits with lipid rafts in a cholesterol-dependent manner.

### V-ATPase Subunits Are Enriched in Type II Cells and Lamellar Bodies

**Figure 3:**
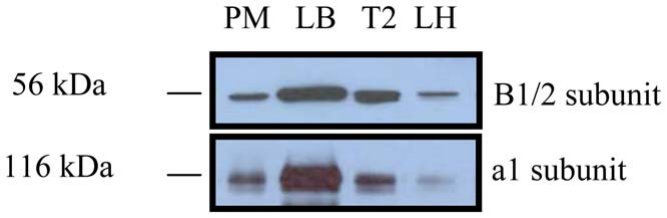
Subcellular localization of V-ATPase subunits. Total proteins isolated from plasma membrane (PM, 20 µg), lamellar bodies (LB, 5 µg), type II cells (T2, 20 µg), and lung tissue homogenates (LH, 20 µg) were immunoblotted for V-ATPase B1/2 and a1 subunits.

We then studied the subcellular localization of V-ATPase subunits. Immunoblotting revealed a much higher amount of B1/2 and a1 subunits in type II cell lysates than that in lung homogenate (Fig. 3). While plasma membrane fractions had a small amount of both subunits, lamellar body fractions contained abundant B1/2 and a1 subunits, indicating that V-ATPases are mainly located on lamellar bodies.

### V-ATPase Inhibition Increases Surfactant Secretion

**Figure 4:**
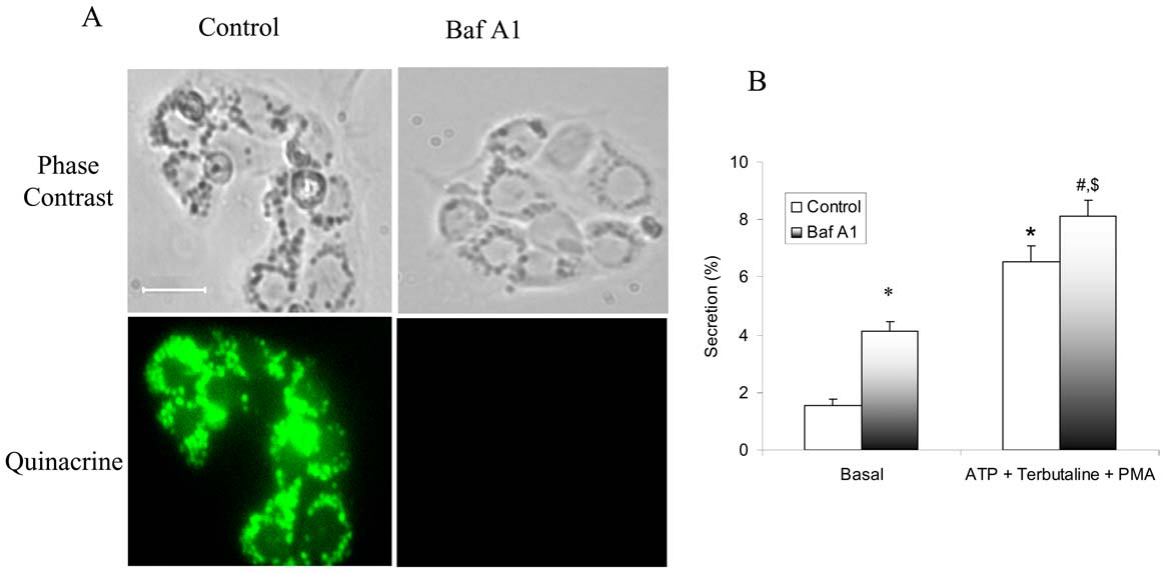
Effect of Bafilomycin A1 on the pH gradient across lamellar body membrane and surfactant secretion. (A) Overnight cultured cells were treated with 20 nM Bafilomycin A1 (Baf A1) for 1 hr. The quinacrine was added to a final concentration of 10 µM. Fluorescence microscopy was undertaken to monitor quinacrine staining. Shown are representative images from 3 independent cell preparations. Scale bar: 10 µm. (B) Freshly isolated type II cells were labeled with [^3^H]choline overnight. The cells were treated with 20 nM Baf A1 for 1 hr, followed by incubation with a combination of 100 µM ATP; 10 µM terbutaline and 0.1 µM PMA for 2 additional hrs. Surfactant secretion was expressed as a percentage of total cellular [^3^H] PC secreted into medium. Data shown are means ± SE (n = 12). *P<0.05 *v.s.* Control (basal); ^#^P<0.05 *v.s.* Baf A1 (basal); ^$^P<0.05 *v.s.* Control (ATP+ Terbutaline +PMA) (ANOVA/Newman-Keuls Multiple Comparison Tests). *Open bars*, control; *shaded bars*, Baf A1.

The acidic milieu in the lamellar bodies is required for the processing of surfactant proteins and packaging of surfactant lipids [26], [27]. However, its role in lung surfactant secretion is unknown. We thus studied if V-ATPases participate in lamellar body exocytosis. We inhibited V-ATPases with Baf A1, a macrolide antibiotic isolated from *Streptomyces griseus* and a specific and potent inhibitor of V-ATPase at nanomole concentrations. We ascertained the inhibition of V-ATPase by monitoring the accumulation of quinacrine [25]. The cells were stained with quinacrine for 1 min at the end of treatments and examined for fluorescence. In the untreated type II cells, quinacrine accumulated in the acidic lamellar bodies. Fluorescence in the cells treated with Baf A1 was lost (Fig. 4A), indicating that the pH gradient across the lamellar body membrane was dissipated.

To investigate the effect of V-ATPase inhibition on surfactant secretion, type II cells were treated with Baf A1 and surfactant secretion was measured. Baf A1 increased surfactant secretion to 4.30±0.29% from 1.75±0.21% in the untreated cells (Fig. 4B). The increase in secretion was not due to deleterious effects as cell viability was unchanged in the Baf A1-treated cells (data not shown). Additionally, the effects of Baf A1 on the stimulated surfactant secretion were also examined. Lung secretagogues were added following a 1-hr treatment of the cells with Baf A1, since by this time the pH gradient was lost. Baf A1 slightly but significantly increased the stimulated surfactant secretion (p<0.05). This is probably because the secretion had reached a saturation level under the stimulated conditions.

### Intracellular Ca^2+^ Participates in the V-ATPase-Mediated Surfactant Secretion

**Figure 5:**
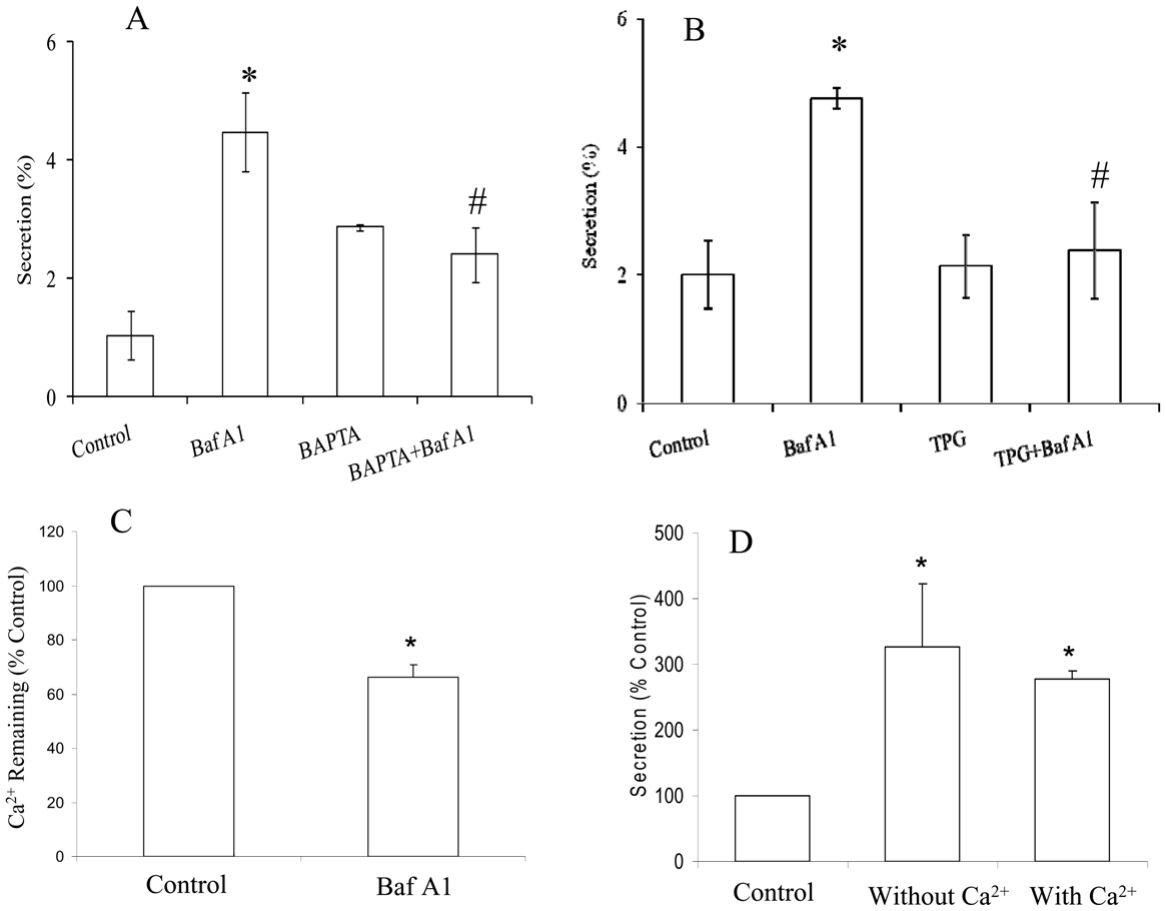
Effects of intracellular Ca^2+^ on V-ATPase-mediated surfactant secretion. (A, B) Overnight cultured type II cells were treated with 20 nM Bafilomycin A1 (Baf A1) in the presence or absence of the intracellular Ca^2+^-chelator, BAPTA-AM (50 µM) (Panel A) or the ER Ca^2+^-ATPase inhibitor, thapsigargin (TPG, 100 µM) (Panel B). Surfactant secretion was expressed as a percentage of total cellular [^3^H] PC secreted into medium. Data shown are means ± SE. *P<0.05 *v.s.* control; #P<0.05 *v.s.* Baf A1 (ANOVA/Newman-Keuls Multiple Comparison Tests, n = 3 independent cell preparations for Panel A and n = 4 independent cell preparations for Panel B). (C) Isolated lamellar bodies were loaded with ^35^Ca^2+^. After treated with 20 nM Baf A1 for 10 min, ^45^Ca retained in lamellar bodies was measured. *P<0.05 *v.s.* control (n = 4 lamellar body preparations). (D) Type II cells were incubated with 20 nM Baf A1 for 1 hr in Ca^2+^-free or 2 mM Ca^2+^ buffer and surfactant secretion was measured. *P<0.01 *v.s.* control (n = 3 independent cell preparations).

In a quest to understand the mechanisms of Baf A1-induced increase in surfactant secretion, we first explored the possibility of an increase in intracellular Ca^2+^ ([Ca^2+^]_i_) as one of the reasons. To this end, type II cells were pre-treated with BAPTA-AM to chelate [Ca^2+^]_i_ and then treated with Baf A1. BAPTA-AM slightly increased secretion consistent with an earlier report [30]. BAPTA-AM significantly decreased Baf A1-stimulated secretion (Fig. 5A), suggesting an involvement of [Ca^2+^]_i_ in the V-ATPase-mediated surfactant secretion.

Lamellar bodies contain a high level of Ca^2+^ and are a potential intracellular storage organelle for exocytosis [31]. Since V-ATPases are mainly located on lamellar bodies, we considered that the inhibition of V-ATPases may result in the release of lamellar body Ca^2+^ and thus increase [Ca^2+^]_i_. To test this possibility, we isolated lamellar bodies and loaded with ^45^Ca^2+^. After being treated with 20 nM Baf A1 for 10 min, ^45^Ca^2+^ retained in lamellar bodies was measured. Baf A1 significantly reduced ^45^Ca^2+^ retained in lamellar bodies (Fig. 5B), indicating that Baf A1 promotes Ca^2+^ release from the lamellar bodies. To see whether Baf A1 affects the integrity of lamellar body membrane, we labeled the lamellar body surfactant lipids in rats for 18 hrs by intraperitoneal injection of [^3^H]choline chloride. The labeled lamellar bodies were isolated and treated with 20 nM Baf A1 for 10 min. There was no difference in radiolabels between controls and Baf A1-treated samples (data not shown), indicating no surfactant was leaked out from the lamellar bodies following Baf A1 treatment.

ER is another Ca^2+^ storage organelle in cells. Ca^2+^-ATPase on the ER membrane is responsible for the accumulation of Ca^2+^. We depleted the ER Ca^2+^ pool by inhibiting the Ca^2+^-ATPase using thapsigargin. There were no differences in secretion following the treatment with thapsigargin when compared to the control (Fig. 5C). However, the Baf A1-mediated secretion was effectively inhibited by thapsigargin. These results indicate that Ca^2+^ cross-talk between ER and lamellar bodies.

An increase in [Ca^2+^]_i_ might be due to mobilization from intracellular pools or entry of Ca^2+^ from extracellular milieu. To determine whether extracellular Ca^2+^ or store-operated channels were involved in the V-ATPase-mediated surfactant secretion, we measured the Baf A1-mediated surfactant secretion in the presence and absence of extracellular Ca^2+^ media. As shown in Fig. 5D, extracellular Ca^2+^ had no effects on the Baf A1-stimulated secretion. Our results suggest that V-ATPase inhibition causes mobilization of Ca^2+^ from intracellular pools (lamellar bodies and ER).

### PKC and CaMKII Are Involved in the V-ATPase-Mediated Surfactant Secretion

Three signaling transduction pathways are involved in stimulated surfactant secretion [32]: (i) Purinoceptor pathway: The activation of this receptor with ATP generates two second messengers IP_3_ and diacylglycerol, which lead to the release of intracellular Ca^2+^ and the activation of PKC; (ii) β-adrenergic receptor pathway: The stimulation of this receptor with terbutaline increases cAMP level and activates PKA; and (iii) Ca^2+^ influx pathway: Ca^2+^ ionophore A23187 stimulates surfactant secretion by raising [Ca^2+^]_i_ and activating CaMKII. The activation of two or more pathways normally results in an additive effect.

**Figure 6:**
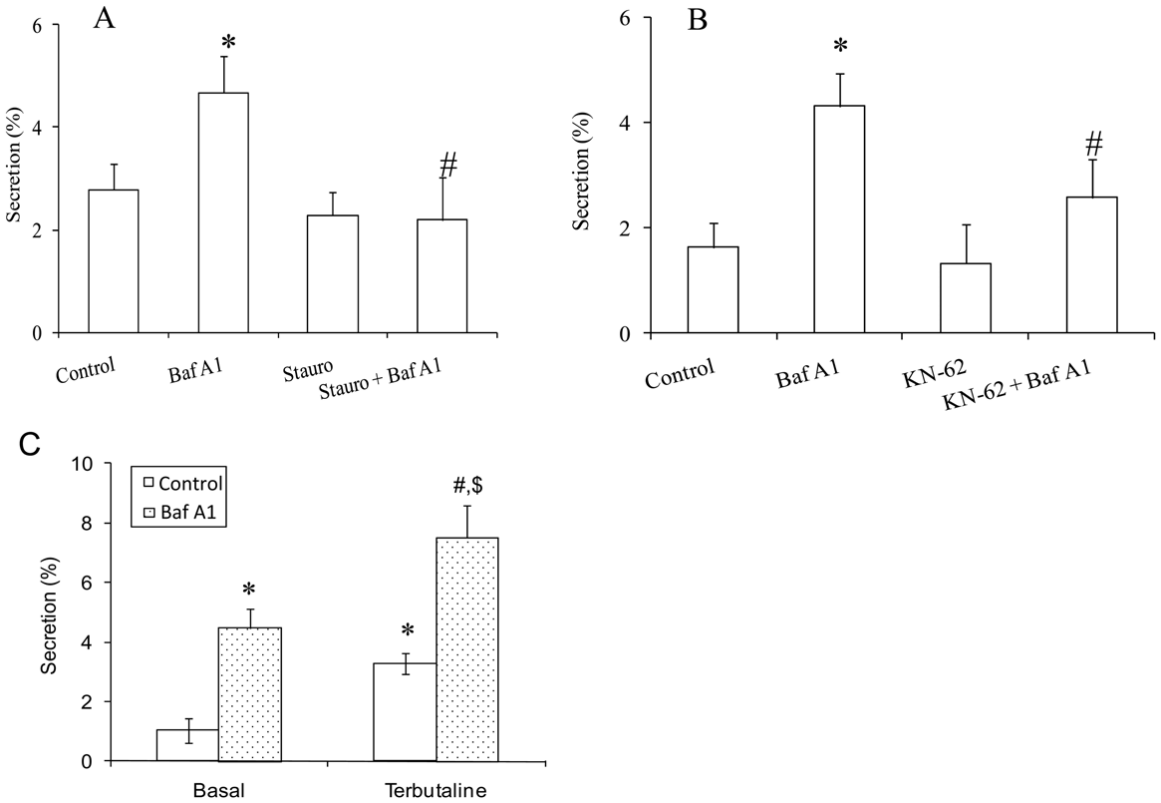
Effects of protein kinase inhibitors on V-ATPase-mediated surfactant secretion. Overnight cultured type II cells were treated with 20 nM Bafilomycin A1 (Baf A1) in the presence or absence of staurosporine (Stauro, 10 nM) (Panel A), KN-62 (10 µM) (Panel B) or 20 µM terbutaline (Panel C). PC secretion was assayed. Data shown are means ± SE. *P<0.05 *v.s.* Control; #P<0.05 *v.s* Baf A1 (ANOVA/Newman-Keuls Multiple Comparison Tests, n = 3 independent cell preparations for Panel A and n = 4 independent cell preparations for Panel B). *P<0.05 *v.s.* Control (basal); ^#^P<0.05 *v.s.* Baf A1 (basal); ^$^P<0.05 *v.s.* Control (Terbutaline) (ANOVA/Newman-Keuls Multiple Comparison Tests, n = 3–4 independent cell preparations for Panel C).

To determine which pathway(s) is involved in the V-ATPase-stimulated surfactant secretion, we examined the effects of PKC and CaMKII inhibitors on the Baf A1-mediated secretion since [Ca^2+^]_i_ is involved in the Baf A1-stimulated surfactant secretion. Staurosporine and KN-62 were used to inhibit PKC and CaMKII, respectively. Both agents effectively inhibited the Baf A1-mediated increase in surfactant secretion (Fig. 6A and 6B). The inhibitors alone had no effect.

If Baf A1 elicits its effect through PKC/CaMKII pathways, an additive effect should be observed when Baf A1 and terbutaline (β2 agonist) were used together. Indeed, the addition of Baf A1 and terbutaline resulted in an additive increase in secretion (Fig. 6C). The result suggests that Baf A1-stimulated secretion is not via the PKA pathway.

### Lung Surfactant Secretagogues Disrupt the pH Gradient Across the Lamellar Body Membrane

**Figure 7:**
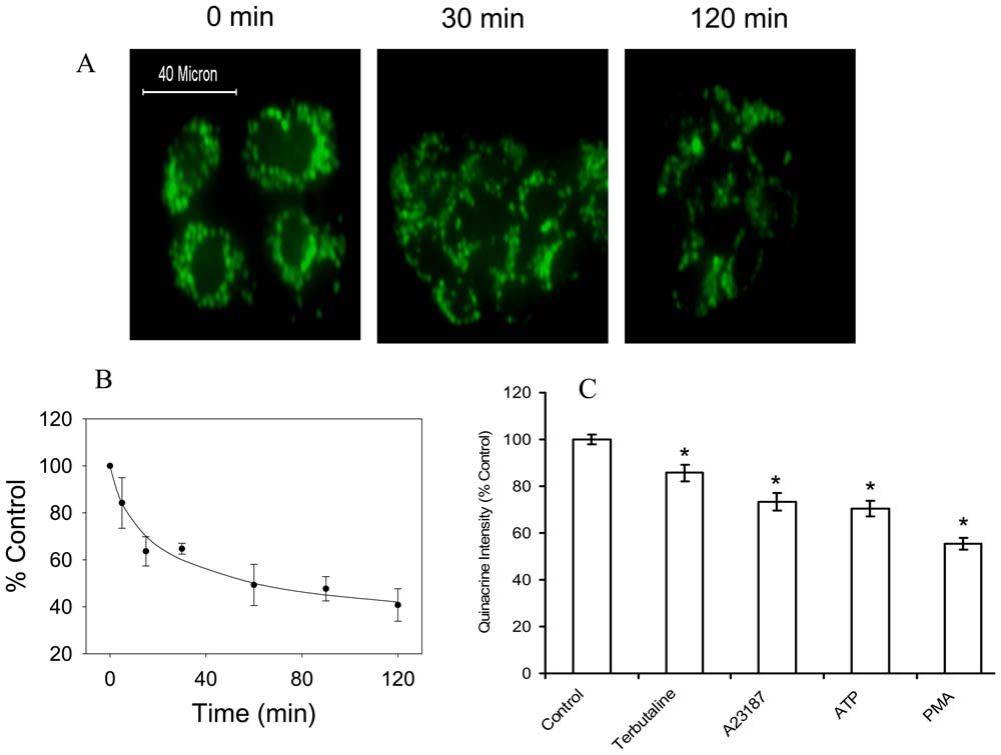
Effect of lung surfactant secretagogues on the lamellar body pH in alveolar type II cells. Overnight cultured type II cells were stimulated with a combination of 200 µM ATP and 0.2 µM PMA for various times indicated in the figure. At the end of incubation, the cells were immediately fixed and examined under a fluorescence microscope. (A) Shown are the representative images indicating the changes in quinacrine accumulation as a function of time. Scale bar: 40 µm. (B) The intensity of quinacrine staining was quantified and expressed as a percentage of control. Shown are means ± SE (n = 3 independent cell preparations). (C) Overnight cultured type II cells were stimulated with 20 µM terbutaline, 0.2 µM A23187, 1 mM ATP, or 0.1 µM PMA for 2 hours. The cells were incubated with quinacrine as above. Quinacrine staining intensity was quantified and expressed as arbitrary units. Shown are means ± SE (n = 3 independent cell preparations).

We asked if there was a physiological relevance of the stimulation to surfactant secretion by V-ATPase inhibition. We examined the effects of lung surfactant secretagogues on the pH gradient across the lamellar body membrane. We stimulated type II cells with a combination of lung surfactant secretagogues (ATP and PMA) for various times and monitored the lamellar body pH by the accumulation of quinacrine. A gradual decrease in accumulation of the dye was observed in the lamellar bodies following the stimulation with secretagogues (Fig. 7A). The quantitation of the fluorescence intensity revealed a ∼50% decrease at 60 min (Fig. 7B). The decrease in fluorescence is unlikely due to the release of quinacrine via exocytosis because type II cells were stained with quinacrine for only about one minute at the end of stimulation. It is also unlikely that a decrease in number of lamellar bodies accounts for the decrease in fluorescence because under the stimulated conditions used, ∼8% of lung surfactant is secreted (Fig. 4B) while the decrease in fluorescence is ∼60%.

We then stimulated type II cells with individual lung secretagogues (Terbutaline, A23187, ATP and PMA) that activates three signaling pathways and examined their effects on lamellar body pH. As shown in Fig. 7C, all the secretgaogues decrease quinacrine staining, suggesting that a decrease in lamellar body pH caused by secretagogues is independent of up-stream signals.

### Disassembly of V-ATPase Complex Following Stimulation with Lung Surfactant Secretagogues

**Figure 8:**
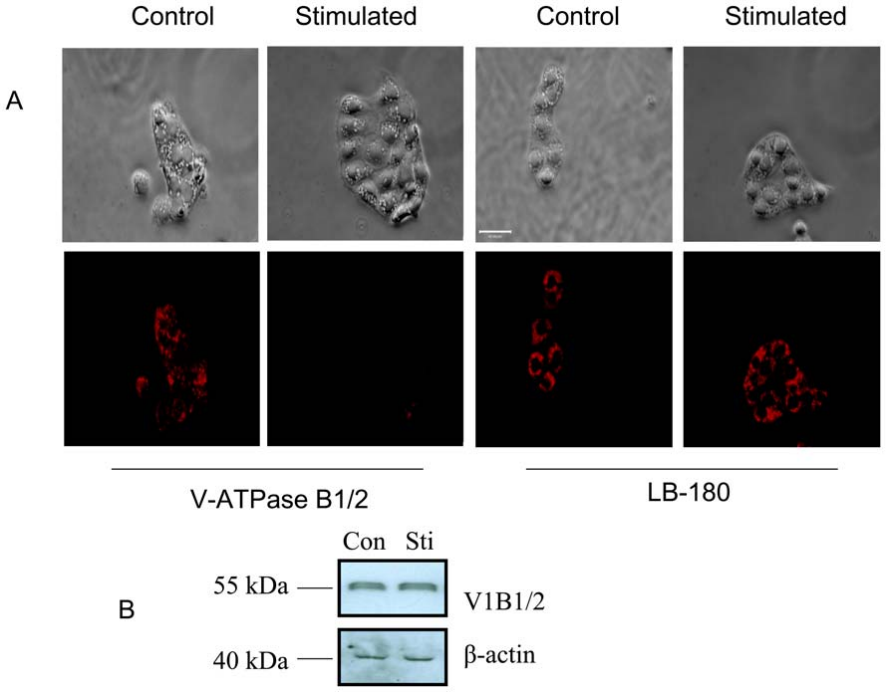
Differential localization of V-ATPase B1/2 subunits following stimulation of cells with lung surfactant secretagogues. (A) Overnight cultured cells were stimulated with a combination of secretagogues 200 µM ATP and 0.2 µM PMA for 2 hrs. Immunostaining was performed to detect the localization of V-ATPase B1/2 subunit. The cells were also stained for the LB-180 protein to check for any changes in the lamellar body membrane following stimulation. Shown are the representative images. Scale bar: 40 µm. (B) Equal amounts of the total proteins (25 µg) isolated from control (Con) and stimulated (Sti) cells as in Panel A were immunobloted for V-ATPase B1/2 subunit and reprobed with β-actin. Shown are the representative immunoblots from 4 independent cell preparations.

An increase in lamellar body pH following stimulation of type II cells with lung surfactant secretagogues suggests that the secretagogues inhibit V-ATPase activity. Since V-ATPase is regulated by the reversible dissociation of the integral Vo and peripheral V1 domains [33], we determined whether the inactivation of V-ATPase by surfactant secretagogues is due to the disassembly of Vo and V1 domains. We chose the B1/2 subunit for V1 domain. The B1/2 subunit antibodies stained lamellar bodies in the control cells, but not the stimulated cells (Fig. 8A), indicating that the B1/2 subunit is dissociated from Vo domain in the stimulated cells. There was no difference in LB-180 (a lamellar body membrane protein) staining between control and stimulated cells. Western blot analysis demonstrates that B1/2 subunit protein levels were the same in the control and stimulated cells (Fig. 8B), indicating that B1/2 subunit was not degraded. The disappearance of the B1/2 immuofluorescence under the stimulated conditions is likely due to the blocking of the epitope on B1/2 subunit after being translocated to cytoplasm from lamellar body membrane.

### Knockdown of V-ATPase a1 and B2 Subunits Leads to Reduced Surfactant Secretion by Type II Cells

**Figure 9:**
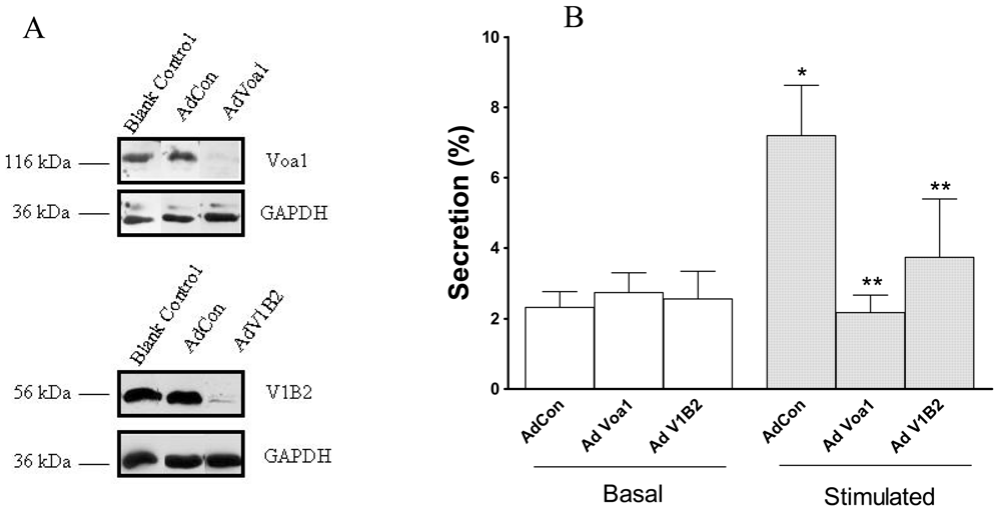
Effect of V-ATPase subunit a1 and B2 knockdown on surfactant secretion by alveolar type II cells. (A) Freshly isolated type II cells were transduced without (Blank Con) and with adenoviruses (MOI: 100) containing irrevelant siRNA sequences (AdCon), or siRNAs targeted to a1 (AdVoa1) and B2 (AdV1B2) subunits for 5 days. Cell lysates were immunoblotted for a1 and B2 proteins. The blots were reprobed for GAPDH for confirming equal loading of protein. (B) Type II cells were cultured on air-liquid cultures system. The cells were transduced with adenoviruses at a MOI of 100 on day 2 and cultured for 5 more days. The cells were labeled with [^3^H]-choline on day 6 overnight, then stimulated with 1 mM ATP for 2 hrs and assayed for surfactant secretion. The results were expressed as a percentage of unstimulated cells transduced with AdCon. Data shown are means ± SE (n = 4 independent cell preparations).

Finally we investigated if V-ATPase subunits were crucial for surfactant secretion. We used RNAi to silence a1 and B2 subunits. Efficient knockdown of a1 and B2 was achieved after 5 days treatment with adenoviral vectors carrying shRNAs targeted to a1 and B2 (Fig. 9A). The control virus had no effects. We then investigated the effects of a1 and B2 subunit knock-down on surfactant secretion. To maintain type II cell phenotype, we used an air-lipid model to culture type II cells [34]. Under the conditions used, the cells cultured for 6 days contain lamellar bodies similar to that seen *in vivo* and secrete surfactant in response to various surfactant secretagogues [34]. We have previously used this model to investigate the effects of the knock-down of flotillin-2 or annexin A2 on surfactant secretion [11], [35]. Nile red selectively stains lamellar bodies in type II cells [36]. There were no differences in the Nile red staining patterns between control and a1 or B2-knocked-down type II cells (data not shown). The knockdown of a1 and B2 subunits had no effects on basal surfactant secretion, but reduced stimulated surfactant secretion (Fig. 9B). The results suggest that V-ATPase is required for stimulated surfactant secretion.

## Discussion

Alveolar type II cells synthesize, store and secrete lung surfactant. Our previous study indicated the role of lipid rafts in surfactant secretion [1]. However, data is lacking on the entire protein components of lipid rafts in type II cells. The present proteomic analysis uncovered the proteins involved in diverse cellular processes in the lipid rafts isolated from type II cells. V-ATPase was a stoichiometrically significant component in the lipid rafts and was studied in detail for its role in surfactant secretion. Our results indicated that the inhibition of V-ATPase stimulated surfactant secretion by releasing Ca^2+^ from lamellar bodies and ER, and activating PKC and CaMKII.

Type II cell lipid raft proteins included those involved in acidification (V-ATPases), metabolism (ATP synthases) and cellular proliferation and differentiation (prohibitin), phospholipid-binding proteins (annexin A2), cytoskeletal proteins (actin), and oxygen exchange (globin beta1). Previous studies have observed many of these proteins in lipid rafts of other cells [12]–[20]; however, our current work focuses on identifying only the most abundant proteins in lipid rafts, rather than an exhaustive catalog of this organelle because of the limitations of the MALDI-TOF technique. Lipid rafts are found not only on plasma membrane, but also in intracellular compartments. For example, our previous studies have shown that the lipid raft marker proteins, flotillin-1 and -2 were present on lamellar bodies, indicating that lipid rafts exist in lamellar bodies [1]. Since different subcellular membranes contain different proteins, it is likely that proteomic profiles in lipid rafts of various subcellular compartments are different. The lipid raft fractions in current studies were isolated from whole type II cell lysate and thus contained mixed lipid rafts from all of the subcellular compartments. The relative contributions of lipid rafts from different subcellular compartments to the total cell lipid rafts remain to be determined. However, the abundance of the lamellar body-enriched enzyme, V-ATPase, in the type II cell lipid rafts suggests that lamellar body lipid rafts may dominate the total type II cell lipid rafts. Some of the identified proteins such as F-ATPase subunit E and beta were of mitochondrial origin. Similar results were also reported in other cell types [12], [14], [20], [37], [38]. It is possible that lipid rafts are also present in mitochondria.

Lung lamellar bodies are lysosomal origin as indicated by the localization of lysosomal associated membrane protein with lamellar body marker protein, ABCA3 [39]. They also contain lysosomal enzymes [40]. Lamellar bodies in type II cells maintain an acidic milieu (pH ∼6.0) due to the V-ATPase activity [25], [41]. The acidic pH was confirmed by the accumulation of dyes, such as quinacrine and acridine orange [25], [27]. Although quinacrine may also stain lysosomes, we believe that the quinacrine staining in type II cells is mainly due to lamellar body staining for the following reasons: (i) The examination of images indicates that the quinacrine staining in type II cells are granular appearance (Fig. 4A), a typical lamellar body staining pattern with lamellar body marker, LB-180 (Fig. 8A); (ii) Although the relative abundance of lamellar bodies and lysosomes in type II cells is unclear, lamellar bodies are likely more than lysosomes since type II cells are main storage cells of lung surfactant; (iii) The staining of type II cells with quinacrine is faster (only a few seconds) than alveolar macrophages (4–5 minutes), which have abundant lysosomes [25].

Acidic pH in the lamellar bodies mediated by V-ATPase is essential for the packaging of surfactant lipids, proteolytic processing of surfactant protein C, and Ca^2+^ uptake [26], [27], [41], [42]. Our current studies revealed that the inhibition of V-ATPase resulted in an increased surfactant secretion, indicating a new functional role of V-ATPase in lung surfactant secretion. Different types of cells respond differently to the V-ATPase inhibition. In PC12 cells, Baf A1 decreases the secretagogue-stimulated secretion of chromagranin A primarily via interfering sorting of the protein and increased the basal secretion [43]. V-ATPase inhibition results in a disruption of acidic pH of secretory granules, but has no effects insulin secretion in pancreatic islet beta cells [44], [45]. However, V-ATPase inhibitors increase secretion in pancreatic alpha cells and glial cell lines [46].

V-ATPases on lamellar bodies generate not only a pH gradient (ΔpH_v_), but also a transvesicular proton-electrochemical potential gradient (ΔΨ_v_).The treatment of type II cells with methylamine and ammonium chloride increases the lamellar body pH and enhances surfactant secretion [27]. However, methylamine and ammonium chloride also increase cytosolic pH transiently. The increase of surfactant secretion by the Baf A1 inhibition of V-ATPase is likely due to an increase in lamellar body pH. It is less likely that Baf A1 affects cytoplasmic pH in type II cells since V-ATPase is highly enriched in lamellar bodies and there is only minimal expression of V-ATPase on plasma membranes of type II cells. However, in some specialized cells such as macrophages and osteoclasts, V-ATPases are highly expressed on plasma membranes. These enzymes may modulate cytoplasmic pH and are responsible for the acidification of these cells [47], [48].

There are several phospholipases A2 (PLA2) in type II cells involved in various aspects of cellular functions. We have previously shown that cytosolic phopholipase A2 regulates surfactant secretion through the generation of arachidonic acid and secretory PLA2 and Ca^2+^-independent PLA2 has no effects [49]. Lysosomal-type PLA2 (aiPLA2) is acidic Ca^2+^-independent PLA2. The enzyme is also named peroxiredoxin 6 and is a dual functional protein with PLA2 and glutathione peroxidase activities [50], [51]. aiPLA2 is enriched in lamellar bodies and shows a maximal activity at pH 4. aiPLA2 plays a role in the degradation of DPPC [52]. This raises a question whether the increase in surfactant secretion by the inhibition of lamellar body V-ATPase simply reflects the inhibition of DPPC degradation, accumulation of DPPC content in the lamellar bodies, and the more DPPC release by the same number of exocytotic events. We do not believe that this is the case since NH_4_Cl or methylamine only reduces the DPPC degradation in type II cells by 20% [52] and under our treatment conditions with Baf A1, we did not observe a significant increase in radiolabels in PC (103±3% of control, n = 11).

Intracellular Ca^2+^ chelation abrogated the Baf A1-induced secretion, indicating a critical role of Ca^2+^ in this process. Intracellular Ca^2+^ can be altered by the mobilization of Ca^2+^ from intracellular or extracellular pools. We did not observe differences in the Baf A1-stimulated surfactant secretion in the absence or presence of extracellular Ca^2+^, which excludes the contribution of extracellular Ca^2+^ to the V-ATPase-mediated surfactant secretion. Lamellar bodies contain mM Ca^2+^ [31] and are one of the potential intracellular sources. Also, the Ca^2+^ concentration in exocytotic lamellar bodies is higher than in perinuclear lamellar bodies. Our present studies demonstrate that the inhibition of V-ATPase promoted the release of Ca^2+^ from isolated lamellar bodies. Dissipation of lysosomal pH in macrophages led to a gradual loss of lysosomal Ca^2+^ with a simultaneous increase in cytosolic levels [53]. A similar process may occur in lamellar bodies since V-ATPase was predominantly present on the lamellar body membrane and its inhibition led to the dissipation of pH gradient.

In addition to lamellar bodies, ER is another source of intracellular Ca^2+^. Depletion of ER Ca^2+^ pools inhibited the V-ATPase-mediated increase in surfactant secretion, indicating the cross-talk between ER and lamellar body Ca^2+^ pools. This is supported by previous reports that small and localized changes in Ca^2+^ induce global Ca^2+^ waves due to interplay between different Ca^2+^ pools. In arterial smooth muscle cells, NAADP increases Ca^2+^ release from Baf A1-sensitive compartment, which in turn further induces Ca^2+^ release from sarcoplasmic reticulum by Ca^2+^-induced Ca^2+^ release owing to the close apposition of these two pools [54]. It is possible that such an apposition may exist in type II cells.

A rise of intracellular Ca^2+^ may activate PKC and CaMKII. When type II cells were treated with PKC and CaMKII inhibitors, we observed an inhibition in the Baf A1-induced surfactant secretion. Similar results were reported for lysosomal secretion in macrophages [55]. The PKA pathway appears not to be involved in the Baf A1-stimulated surfactant secretion since the effects of Baf A1 and terbutaline were additive. Our results thus support the idea that the V-ATPase-mediated surfactant secretion is mediated by PKC and CaMKII.

The finding that the inactivation of V-ATPase by Baf A1 stimulates surfactant secretion raises a question: does this process occur at physiological conditions? Dissipation of the pH gradient across the lamellar body membrane by lung surfactant secretagogues suggests that V-ATPase is inactivated during the stimulation of type II cells for secretion, which may be part of signal transduction pathways for surfactant secretion. Earlier studies have indicated that the reversible dissociation of Vo and V1 subunits is one of the mechanisms by which V-ATPase activity is regulated [33]. Our results showed that the stimulation of type II cells with lung surfactant secretagogues led to the disappearance of the V-ATPase B1/2 subunit (a subunit in the peripheral V1 domain). This is not due to the degradation of B1/2 subunits since B1/2 protein levels were not changed as determined by Western blot analysis. Most likely, the B1/2 subunits were translocated from lamellar body membrane to cytoplasm due to the disassembly of Vo and V1 domains. The disappearance of B1/2 subunits is probably because of the blocking of the epitope recognized by the antibody. The disassembly of Vo and V1 domains upon stimulation have been observed in yeast and some mammalian cells [56], [57]. The mechanisms for lung surfactant secretagogue-induced disassembly of Vo and V1 domains remain to be determined. Some of the V-ATPase subunits have been shown to be phosphorylated by various kinases [58]–[60]. It is possible that the inactivation of V-ATPase by lung surfactant secretagogues involves the phosphorylation of V-ATPase subunits.

Knockdown of V-ATPase a1 and B2 subunits resulted in reduced stimulated surfactant secretion by type II cells. These results suggest that V-ATPases are necessary for stimulated surfactant secretion. Although we did not observe significant differences in lipid staining of lamellar bodies with Nile red between control and the knocked-down cells, we cannot rule out the possibility that the accumulation of lipids in lamellar bodies are impaired due to the knock-down of V-ATPase subunits since the acidic pH is required for the packaging of surfactant lipids [27]. V-ATPases are known to be involved in exocytosis at various stages of this process. The secretion of insulin is reduced in a3-subunit knock-out mice [45]. In flies, mutations in vha 100-1 (a subunit) lead to a defect in vesicular exocytosis [61]. In yeast, integral membrane Vo subunits form a trans-complex, resulting in a proteolipid pore, which is required for membrane fusion [62], [63]. Several V-ATPase subunits also interact with SNARE proteins in a number of cell systems [61], [64]. Knockdown of these subunits might have contributed to loss of their interactions, thus inhibiting membrane fusion.

Earlier studies have indicated that ventilating the lungs with air containing low CO_2_ results in an increase in surfactant secretion. The increase is attributed to cellular alkalosis and is independent of β-adrenergic pathway [65]. These changes in CO_2_ concentrations mimic physiological and clinical conditions such as swimming and hyperventilation and have been shown to result in respiratory alkalosis [66], [67]. Therefore, our finding on the enhancement of surfactant secretion by increased lamellar body pH via V-ATPase may be of physiological relevance.

**Figure 10:**
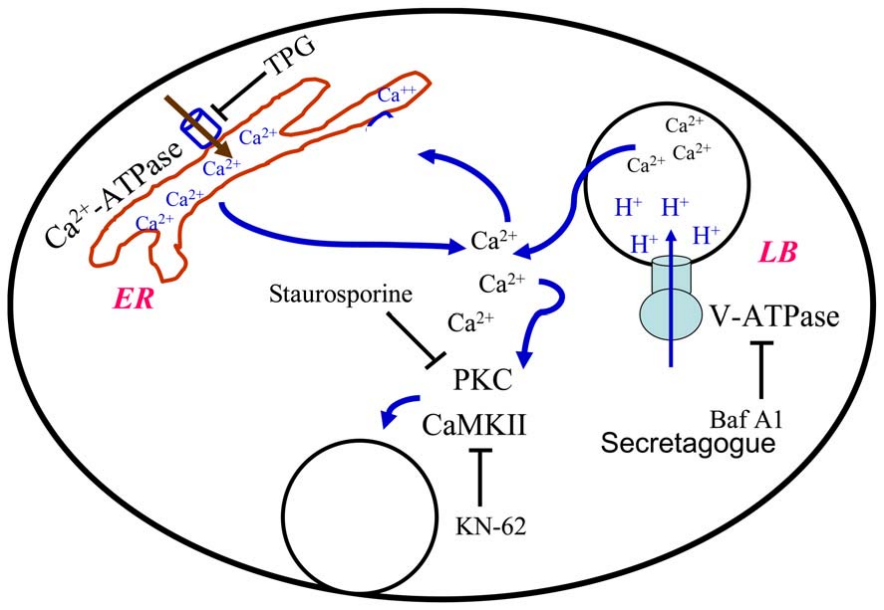
Schematic representation of proposed events for V-ATPase-mediated lung surfactant secretion. Following V-ATPase inhibition by Baf A1 or lung surfactant secretagogues, Ca^2+^ is mobilized from lamellar bodies. The small localized changes in Ca^2+^ concentration leads to the further release of Ca^2+^ from ER store. The global increase in intracellular Ca^2+^ concentration results in the activation of PKC and CaMKII and the increase in surfactant secretion. LB: lamellar bodies; ER: endoplasmic reticulum.

In summary, we propose the following model for V-ATPase-mediated surfactant secretion (Fig. 10). Lung surfactant secretagogues inactivate V-ATPases on lamellar bodies and dissipate the lamellar body pH gradient. The increase in the lamellar body pH results in the mobilization of Ca^2+^ from lamellar bodies, leading to an increase in cytosolic free Ca^2+^. Such an increase in Ca^2+^ further releases Ca^2+^ from the ER pool. The net surge on Ca^2+^ concentration activates PKC and CaMKII and finally increases lung surfactant secretion. The current studies for the first time demonstrate that V-ATPase modulates surfactant secretion via lamellar body and ER Ca^2+^ pools.

## References

[1] Chintagari NR, Jin N, Wang P, Narasaraju TA, Chen J (2006). Effect of cholesterol depletion on exocytosis of alveolar type II cells.. Am J Respir Cell Mol Biol.

[2] Salaun C, James DJ, Chamberlain LH (2004). Lipid rafts and the regulation of exocytosis.. Traffic.

[3] Pelkmans L, Helenius A (2002). Endocytosis via caveolae.. Traffic.

[4] Allen JA, Halverson-Tamboli RA, Rasenick MM (2007). Lipid raft microdomains and neurotransmitter signalling.. Nat Rev Neurosci.

[5] Schuck S, Simons K (2004). Polarized sorting in epithelial cells: raft clustering and the biogenesis of the apical membrane.. J Cell Sci.

[6] Zaas DW, Duncan M, Rae WJ, Abraham SN (2005). The role of lipid rafts in the pathogenesis of bacterial infections.. Biochim Biophys Acta.

[7] Takeda M, Leser GP, Russell CJ, Lamb RA (2003). Influenza virus hemagglutinin concentrates in lipid raft microdomains for efficient viral fusion.. Proc Natl Acad Sci U S A.

[8] Cordy JM, Hooper NM, Turner AJ (2006). The involvement of lipid rafts in Alzheimer's disease.. Mol Membr Biol.

[9] Abonyo BO, Wang P, Narasaraju TA, Rowan WH, McMillan DH (2003). Characterization of alpha-Soluble N-Ethylmaleimide-Sensitive Fusion Attachment Protein in Alveolar Type II Cells: Implications in Lung Surfactant Secretion.. Am J Respir Cell Mol Biol.

[10] Abonyo BO, Gou D, Wang P, Narasaraju T, Wang Z (2004). Syntaxin 2 and SNAP-23 are required for regulated surfactant secretion.. Biochemistry.

[11] Chintagari NR, Gou D, Liu L (2008). Knockdown of flotillin-2 inhibits lung surfactant secretion by alveolar type II cells.. Cell Res.

[12] Bini L, Pacini S, Liberatori S, Valensin S, Pellegrini M (2003). Extensive temporally regulated reorganization of the lipid raft proteome following T-cell antigen receptor triggering.. Biochem J.

[13] Razzaq TM, Ozegbe P, Jury EC, Sembi P, Blackwell NM (2004). Regulation of T-cell receptor signalling by membrane microdomains.. Immunology.

[14] Sprenger RR, Horrevoets AJ (2007). Proteomic study of caveolae and rafts isolated from human endothelial cells.. Methods Mol Biol.

[15] Sleight SB, Miranda PV, Plaskett NW, Maier B, Lysiak J (2005). Isolation and proteomic analysis of mouse sperm detergent-resistant membrane fractions: evidence for dissociation of lipid rafts during capacitation.. Biol Reprod.

[16] MacLellan DL, Steen H, Adam RM, Garlick M, Zurakowski D (2005). A quantitative proteomic analysis of growth factor-induced compositional changes in lipid rafts of human smooth muscle cells.. Proteomics.

[17] Nguyen HT, Amine AB, Lafitte D, Waheed AA, Nicoletti C (2006). Proteomic characterization of lipid rafts markers from the rat intestinal brush border.. Biochem Biophys Res Commun.

[18] Berkane AA, Nguyen HT, Tranchida F, Waheed AA, Deyris V (2007). Proteomic of lipid rafts in the exocrine pancreas from diet-induced obese rats.. Biochem Biophys Res Commun.

[19] Yanagida M, Nakayama H, Yoshizaki F, Fujimura T, Takamori K (2007). Proteomic analysis of plasma membrane lipid rafts of HL-60 cells.. Proteomics.

[20] Foster LJ, de Hoog CL, Mann M (2003). Unbiased quantitative proteomics of lipid rafts reveals high specificity for signaling factors.. Proc Natl Acad Sci U S A.

[21] Nishi T, Forgac M (2002). The vacuolar (H+)-ATPases–nature's most versatile proton pumps.. Nat Rev Mol Cell Biol.

[22] Karet FE, Finberg KE, Nelson RD, Nayir A, Mocan H (1999). Mutations in the gene encoding B1 subunit of H+-ATPase cause renal tubular acidosis with sensorineural deafness.. Nat Genet.

[23] Frattini A, Orchard PJ, Sobacchi C, Giliani S, Abinun M (2000). Defects in TCIRG1 subunit of the vacuolar proton pump are responsible for a subset of human autosomal recessive osteopetrosis.. Nat Genet.

[24] Smith AN, Skaug J, Choate KA, Nayir A, Bakkaloglu A (2000). Mutations in ATP6N1B, encoding a new kidney vacuolar proton pump 116-kD subunit, cause recessive distal renal tubular acidosis with preserved hearing.. Nat Genet.

[25] Chander A, Johnson RG, Reicherter J, Fisher AB (1986). Lung lamellar bodies maintain an acidic internal pH.. J Biol Chem.

[26] Beers MF (1996). Inhibition of cellular processing of surfactant protein C by drugs affecting intracellular pH gradients.. J Biol Chem.

[27] Chander A, Sen N, Wu AM, Higgins S, Wadsworth S (1996). Methylamine decreases trafficking and packaging of newly synthesized phosphatidylcholine in lamellar bodies in alveolar type II cells.. Biochem J.

[28] Jones PG, Fitzpatrick S, Waisman DM (1994). Chromaffin granules release calcium on contact with annexin VI: implications for exocytosis.. Biochemistry.

[29] Gou D, Weng T, Wang Y, Wang Z, Zhang H (2007). A novel approach for the construction of multiple shRNA expression vectors.. J Gene Med.

[30] Strayer DS, Hoek JB, Thomas AP, White MK (1999). Cellular activation by Ca2+ release from stores in the endoplasmic reticulum but not by increased free Ca2+ in the cytosol.. Biochem J.

[31] Eckenhoff RG, Somlyo AP (1988). Rat lung type II cell and lamellar body: elemental composition in situ.. Am J Physiol.

[32] Andreeva AV, Kutuzov MA, Voyno-Yasenetskaya TA (2007). Regulation of Surfactant Secretion in Alveolar Type II cells.. Am J Physiol Lung Cell Mol Physiol.

[33] Breton S, Brown D (2007). New insights into the regulation of V-ATPase-dependent proton secretion.. Am J Physiol Renal Physiol.

[34] Mason RJ, Lewis MC, Edeen KE, McCormick-Shannon K, Nielsen LD (2002). Maintenance of surfactant protein A and D secretion by rat alveolar type II cells in vitro.. Am J Physiol Lung Cell Mol Physiol.

[35] Gou D, Wang P, Jin N, Liu L (2004). Silencing of annexin II in primary culture of alveolar epithelial type II cells.. Annexins.

[36] Liu L, Wang M, Fisher AB, Zimmerman UJP (1996). Involvement of annexin II in exocytosis of lamellar bodies from alveolar epithelial type II cells.. Am J Physiol.

[37] Li N, Shaw AR, Zhang N, Mak A, Li L (2004). Lipid raft proteomics: analysis of in-solution digest of sodium dodecyl sulfate-solubilized lipid raft proteins by liquid chromatography-matrix-assisted laser desorption/ionization tandem mass spectrometry.. Proteomics.

[38] Bae TJ, Kim MS, Kim JW, Kim BW, Choo HJ (2004). Lipid raft proteome reveals ATP synthase complex in the cell surface.. Proteomics.

[39] Salaun B, de Saint-Vis B, Pacheco N, Pacheco Y, Riesler A (2004). CD208/dendritic cell-lysosomal associated membrane protein is a marker of normal and transformed type II pneumocytes.. Am J Pathol.

[40] Weaver TE, Na CL, Stahlman M (2002). Biogenesis of lamellar bodies, lysosome-related organelles involved in storage and secretion of pulmonary surfactant.. Semin Cell Dev Biol.

[41] Wadsworth SJ, Spitzer AR, Chander A (1997). Ionic regulation of proton chemical (pH) and electrical gradients in lung lamellar bodies.. Am J Physiol.

[42] Chander A, Fisher AB, Strauss JF (1982). Role of an acidic compartment in synthesis of disaturated phosphatidylcholine by rat granular pneumocytes.. Biochem J.

[43] Taupenot L, Harper KL, O'Connor DT (2005). Role of H+-ATPase-mediated acidification in sorting and release of the regulated secretory protein chromogranin A: evidence for a vesiculogenic function.. J Biol Chem.

[44] Yamada H, Otsuka M, Hayashi M, Nakatsuka S, Hamaguchi K (2001). Ca2+-dependent exocytosis of L-glutamate by alphaTC6, clonal mouse pancreatic alpha-cells.. Diabetes.

[45] Sun-Wada GH, Toyomura T, Murata Y, Yamamoto A, Futai M (2006). The a3 isoform of V-ATPase regulates insulin secretion from pancreatic beta-cells.. J Cell Sci.

[46] Nishiguchi M, Tokugawa K, Yamamoto K, Akama T, Nozawa Y (2003). Increase in secretion of glial cell line-derived neurotrophic factor from glial cell lines by inhibitors of vacuolar ATPase.. Neurochem Int.

[47] Nordstrom T, Rotstein OD, Romanek R, Asotra S, Heersche JN (1995). Regulation of cytoplasmic pH in osteoclasts. Contribution of proton pumps and a proton-selective conductance.. J Biol Chem.

[48] Brisseau GF, Grinstein S, Hackam DJ, Nordstrom T, Manolson MF (1996). Interleukin-1 increases vacuolar-type H+-ATPase activity in murine peritoneal macrophages.. J Biol Chem.

[49] Liu L (1999). Regulation of lung surfactant secretion by phospholipase A_2_.. J Cell Biochem.

[50] Fisher AB, Dodia C, Yu K, Manevich Y, Feinstein SI (2006). Lung phospholipid metabolism in transgenic mice overexpressing peroxiredoxin 6.. Biochim Biophys Acta.

[51] Fisher AB, Dodia C, Feinstein SI, Ho YS (2005). Altered lung phospholipid metabolism in mice with targeted deletion of lysosomal-type phospholipase A2.. J Lipid Res.

[52] Fisher AB, Dodia C (1996). Role of phospholipase A2 enzymes in degradation of dipalmitoylphosphatidylcholine by granular pneumocytes.. J Lipid Res.

[53] Christensen KA, Myers JT, Swanson JA (2002). pH-dependent regulation of lysosomal calcium in macrophages.. J Cell Sci.

[54] Kinnear NP, Boittin FX, Thomas JM, Galione A, Evans AM (2004). Lysosome-sarcoplasmic reticulum junctions. A trigger zone for calcium signaling by nicotinic acid adenine dinucleotide phosphate and endothelin-1.. J Biol Chem.

[55] Sundler R (1997). Lysosomal and cytosolic pH as regulators of exocytosis in mouse macrophages.. Acta Physiol Scand.

[56] Parra KJ, Kane PM (1998). Reversible association between the V1 and V0 domains of yeast vacuolar H+-ATPase is an unconventional glucose-induced effect.. Mol Cell Biol.

[57] Sautin YY, Lu M, Gaugler A, Zhang L, Gluck SL (2005). Phosphatidylinositol 3-kinase-mediated effects of glucose on vacuolar H+-ATPase assembly, translocation, and acidification of intracellular compartments in renal epithelial cells.. Mol Cell Biol.

[58] Voss M, Vitavska O, Walz B, Wieczorek H, Baumann O (2007). Stimulus-induced phosphorylation of V-ATPase by protein kinase A.. J Biol Chem.

[59] Hong-Hermesdorf A, Brux A, Gruber A, Gruber G, Schumacher K (2006). A WNK kinase binds and phosphorylates V-ATPase subunit C.. FEBS Lett.

[60] Myers M, Forgac M (1993). The coated vesicle vacuolar (H+)-ATPase associates with and is phosphorylated by the 50-kDa polypeptide of the clathrin assembly protein AP-2.. J Biol Chem.

[61] Hiesinger PR, Fayyazuddin A, Mehta SQ, Rosenmund T, Schulze KL (2005). The v-ATPase V0 subunit a1 is required for a late step in synaptic vesicle exocytosis in Drosophila.. Cell.

[62] Morel N, Dunant Y, Israel M (2001). Neurotransmitter release through the V0 sector of V-ATPase.. J Neurochem.

[63] Peters C, Bayer MJ, Buhler S, Andersen JS, Mann M (2001). Trans-complex formation by proteolipid channels in the terminal phase of membrane fusion.. Nature.

[64] Galli T, McPherson PS, De Camilli P (1996). The V0 sector of the V-ATPase, synaptobrevin, and synaptophysin are associated on synaptic vesicles in a Triton X-100-resistant, freeze-thawing sensitive, complex.. J Biol Chem.

[65] Chander A (1989). Regulation of lung surfactant secretion by intracellular pH.. Am J Physiol.

[66] Nicholas TE, Barr HA (1983). The release of surfactant in rat lung by brief periods of hyperventilation.. Respir Physiol.

[67] Hildebran JN, Goerke J, Clements JA (1981). Surfactant release in excised rat lung is stimulated by air inflation.. J Appl Physiol.

